# Acute Effect of Inspiratory Muscle Training on Peripheral Tissue Oxygenation Behavior in Individuals With COPD: A Randomized Crossover Study

**DOI:** 10.1002/pri.70177

**Published:** 2026-02-25

**Authors:** Natália Mota da Silva Borges, Dyego Tavares de Lima, Arthur Bruno de Abreu Morais, Laura Natália Freitas Cavalcante Tenório, Sandyelle Teixeira Vieira, Hellen Maria Lacerda de Oliveira Carneiro, Annicia Lins Freitas, Daniele Ferreira de Santana Silva, Elysson da Silva, Maria Anita Oliveira Souza Paiva, José Vinicius Bulhões da Silva, Kauane Paulino Guedes, Tatiana Onofre Gama, Ana Tereza do Nascimento Sales Figueiredo Fernandes, Ingrid Guerra Azevedo, Pollyana Soares de Abreu Morais, Danielle Aparecida Gomes Pereira, Rafaela Pedrosa, José Heriston de Morais Lima, Eduardo Eriko Tenório de França

**Affiliations:** ^1^ Postgraduate Program in Physical Therapy Universidade Federal da Paraíba João Pessoa Paraíba Brazil; ^2^ Department of Physical Therapy Universidade Federal da Paraíba João Pessoa Paraíba Brazil; ^3^ Department of Physiotherapy State University of Paraíba Campina Grande Paraíba Brazil; ^4^ Department of Therapeutic Processes Universidad Católica de Temuco—La Araucanía–Chile Temuco Chile; ^5^ Department of Physiotherapy João Pessoa University Center ‐ UNIPÊ João Pessoa Paraíba Brazil; ^6^ Department of Physiotherapy Federal University of Minas Gerais Belo Horizonte Minas Gerais Brazil

**Keywords:** breathing exercises, COPD, muscle fatigue, near‐infrared, respiratory muscles, spectroscopy

## Abstract

**Background and Purpose:**

Chronic Obstructive Pulmonary Disease (COPD) is a pulmonary condition characterized by airflow obstruction, which progresses with systemic alterations such as changes in muscle composition and metabolism, anticipating the activation of the inspiratory metaboreflex. This study aimed to analyze the acute effects of Inspiratory Muscle Training (IMT) on peripheral muscle metabolism in individuals with COPD, using near‐infrared spectroscopy (NIRS).

**Methods:**

This randomized, blinded, crossover study included 29 individuals with COPD who underwent three distinct sessions: high‐load IMT (IMT‐Strength, 60% of maximal inspiratory pressure—MIP), low‐load IMT (IMT‐Endurance, 30% of MIP), and a sham protocol. Tissue oxygenation of the gastrocnemius muscle was assessed using NIRS before and after each protocol.

**Results:**

Differences in mean final tissue oxygen saturation were observed only during the IMT‐Endurance protocol. The oxygen desaturation time was shorter during the IMT‐Strength protocol compared with the other groups. Although not statistically significant, patients with more severe COPD (GOLD 3–4) exhibited an oxygen desaturation rate higher during the strength IMT compared with the endurance and sham protocols.

**Conclusions:**

Acute high‐intensity IMT may accentuate the reduction in peripheral perfusion, especially in patients with advanced COPD, suggesting possible metaboreflex activation. Conversely, endurance IMT may improve peripheral perfusion. These findings reinforce the need for careful and individualized prescription of IMT in the COPD population.

**Trial Registration:**

Clinical Trials number: NCT 06827379 https://clinicaltrials.gov/study/NCT06827379

## 1 Introduction

Chronic Obstructive Pulmonary Disease (COPD) is a lung condition characterized by persistent airflow limitation and presents with heterogeneous clinical manifestations of symptoms attributed to abnormalities of the airways and alveoli. It is primarily triggered by exposure to environmental factors, resulting from the gene‐environment interaction throughout an individual's life (GOLD, 2025). In the global context, it is considered one of the top three causes of death. In this scenario, individuals often live with the disease and its complications for years, frequently resulting in premature death. Consequently, COPD represents a major public health challenge, requiring increased healthcare resources. Due to the aging population and continued exposure to risk factors, an increase in COPD cases is predicted (GOLD, 2025).

It is important to emphasize that the affected population presents changes that limit lung emptying during forced expiration, decrease the forced expiratory volume in the first second (FEV1) and the FEV1/forced vital capacity (FVC) ratio, and contribute to gas trapping and pulmonary hyperinflation (GOLD, 2025). This impairs respiratory mechanics, as it contributes to the deterioration of the contractile properties of the inspiratory muscles, causing dyspnea on exertion and limiting exercise capacity (GOLD, 2025).

In addition to the effects on the pulmonary system, COPD implies impairments in other systems, as there is an increased demand on the inspiratory muscles, altering the metabolism of the cardiovascular system and peripheral muscle metabolism (Chan et al. 2023). Individuals with COPD have a reduction in type I fibers, which are rich in mitochondria, leading to a loss of oxidative capacity in their peripheral musculoskeletal system. Several mechanisms may contribute to these changes in muscle metabolic activity, including reduced oxygen delivery to the muscles and impaired oxidative metabolism. These alterations exacerbate metabolite accumulation and subsequently activate the metaboreflex (Kerti et al. 2018; Neunhäuserer et al. 2021; Mota et al. 2023).

Studies by Sheel et al. (2001) demonstrated that isolated loading of the inspiratory muscles, through high inspiratory resistance, was sufficient to induce reflex vasoconstriction in the lower limbs even at rest, indicating that the respiratory metaboreflex does not depend on peripheral muscle contraction. Similar findings were reported by ST CROIX et al. (2000), who demonstrated an increase in muscle sympathetic activity during isolated respiratory muscle fatigue.

Therefore, aiming to improve the quality of life for this population, treatment protocols have been studied to increase physical conditioning, with a more effective response to exercise and a reduction in episodes of dyspnea on exertion (Mota et al. 2023). Inspiratory muscle training (IMT) is a strategy that, according to Vilaça et al. (2019), promotes significant improvements in peripheral muscle function, partly by modulating the metaboreflex and improving ventilatory efficiency, which can decrease the severity of hypoxemia during exercise and consequently reduce the accumulation of metabolites in peripheral muscles. This reduction in metaboreflex activation from prolonged IMT use can decrease the excessive cardiovascular response during exercise, improving exercise tolerance and reducing the perception of effort in individuals with COPD.

In this context, there are still no studies in the literature that evaluate the acute effects of IMT with different loads on peripheral muscle oxygenation in this population. Near‐Infrared Spectroscopy (NIRS) stands out as a non‐invasive evaluation method and can be an important tool to assess the effects of IMT on the oxidative capacity of peripheral muscles. This strategy has proven useful in clinical contexts, as it allows for the continuous, real‐time measurement of tissue oxygenation levels, representing the tissue oxygen saturation in the local capillary bed (Kerget et al. 2024). In this regard, the primary objective of this study is to analyze the acute effects of IMT on peripheral tissue oxygenation using NIRS in individuals with COPD. The secondary objective is to analyze the oxygen desaturation rate according to GOLD severity groups.

## 2 Methods

### 2.1 Study Design and Participants

This was a randomized crossover study with blinded outcome assessment and statistical analysis. This study was conducted at the Cardiorespiratory Research Physiotherapy Laboratory of the Federal University of Paraíba (FUPB) in João Pessoa, Paraíba, in partnership with the Laboratory for Assessment and Research in Cardiorespiratory Performance of the Federal University of Minas Gerais.

Participants were recruited during their routine clinical visits to the pulmonology service at the Lauro Wanderley University Hospital or through spontaneous demand. Invitational posters with information about the research were placed in high‐circulation areas of FUPB, and recruitment was also conducted via social media.

### 2.2 Sample Size Calculation, Randomization, Allocation Concealment, Blinding, and Washout

For the sample size calculation (*n*) of this study, the previous work by Layec et al. (2017) was used as a reference. This study investigated the physiological mechanisms contributing to the delayed recovery of muscle fatigue in patients with COPD after plantar flexion exercise. The variables of interest were the mean and standard deviation (± SD) of the HHb recovery time constant and the HHb mean response time, both obtained using near‐infrared spectroscopy (NIRS) in the triceps surae.

The sample size calculation was performed with G*Power software version 3.1.9.7, considering a repeated‐measures ANOVA for a single group evaluated at three time points, with an effect size (Hedges' g) of 0.29 calculated using the Social Science Statistics software based on the mean ± SD of the HHb time variables reported in the study. A statistical power of 80% and a significance level of 5% were assumed. To ensure robustness of the results and to compensate for potential dropouts, a 20% increase was applied to the initial sample size of 21 participants, resulting in a final sample size of 24 participants.

**FIGURE 1:**
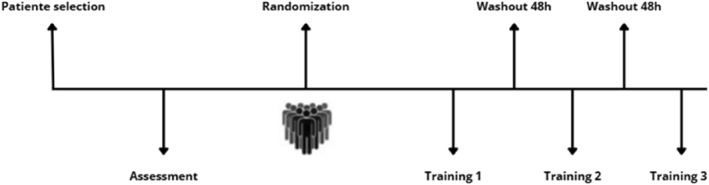
Study design.

All participants underwent three different interventions: strength‐based IMT, endurance‐based IMT, and sham, with a minimum interval of 48 h between sessions, a period considered sufficient to prevent any residual effects (carry over) of IMT (Kuhn et al. 2024). Participants were randomly assigned to one of the three groups using a random number table generated by the website www.random.org. To ensure allocation concealment, opaque sealed envelopes were used. Two independent physiotherapists, who were not involved in the randomization process, conducted the assessments and intervention protocols (Figure 1).

### 2.3 Ethical Aspects

This research project followed the recommendations of the Standard Protocol Items: Recommendations for Interventional Trials (SPIRIT 2013) (Chan et al. 2013) and was conducted in accordance with the Consolidated Standards of Reporting Trials (CONSORT) guidelines (Schulz et al. 2010). This clinical trial was registered at ClinicalTrials.gov (Identifier: NCT 06827379). Participation in the trial was voluntary, through the signing of a Free and Informed Consent Form, duly prepared in accordance with Resolution No. 466/12 of the National Health Council, which deals with guidelines and norms for research involving human beings.

The inclusion criteria were: patients with COPD according to the GOLD classification; age 40 years or older and providing oral and written consent. Participants were excluded if they had inability to perform the IMT protocol and/or functional tests; presence of psychiatric disorders or cognitive and progressive neurological disorders; cognitive impairment or inability to understand commands; individuals using long‐term oxygen therapy (LTOT); COPD exacerbation within the last month before inclusion in the research. Patients who agreed to participate and met the eligibility criteria received an explanation of the study protocol and were invited to consent to participate in the study.

### 2.4 Assessment

#### 2.4.1 Clinical, Demographic, and Anthropometric Data

To collect the sociodemographic and clinical data of the participants, an evaluation form prepared by the researchers of the responsible laboratory was used. The form included anamnesis data such as: personal and demographic information, anthropometric measurements (weight and height), medical history, presence of comorbidities, and medication use. To characterize the sample, participants underwent an evaluation of pulmonary function, respiratory muscle function, and functional capacity.

#### 2.4.2 Pulmonary Function

Spirometry was performed following the criteria of the American Thoracic Society (ATS, 2002). All patients performed at least three maneuvers in a seated position using a nose clip and mouthpiece. They were instructed to perform a maximal inspiration to total lung capacity, followed by a maximal and continuous forced expiration for at least six seconds, until residual volume. The FEV1, FVC, FEV1/FVC ratio, and peak expiratory flow (PEF) were determined.

#### 2.4.3 Inspiratory Muscle Function

To evaluate respiratory muscle function, a computerized electronic device (KH2; PowerBreathe International Ltd., UK) along with a Breathelink feedback system was used. The maximum inspiratory pressure (MIP) and the endurance of the respiratory muscles were assessed. This assessment was conducted with three on a maximum of eight repetitions, seeking variations of less than 10% between values, with the highest value being considered. Respiratory muscle endurance was measured by an incremental load test where patients were instructed to perform the maximum number of breaths. An initial load of 10 cmH_2_O was standardized for a two‐minute period. Subsequently, the patient was instructed to rest for one minute, and then a new two‐minute cycle was initiated, adding another 10 cmH_2_O to the equipment's load. The highest load sustained for at least 1 min was considered the sustained MIP value.

#### 2.4.4 Functional Capacity

Functional capacity was assessed using the 6MWT, which was performed in a flat 30‐m corridor where the individual was instructed to walk at a comfortable pace, without running, for a period of 6 min. During the test, variables such as heart rate (HR), peripheral oxygen saturation (SpO_2_), blood pressure (BP), and the modified Borg scale of perceived exertion were measured before and at the end of the test. HR and SpO_2_ were monitored throughout the test. At the end of the test, the maximum distance covered in meters was recorded.

### 2.5 Intervention

After randomization, participants were allocated to one of three groups: strength‐based IMT, endurance‐based IMT, or sham, using the KH2 device (PowerBreathe International Ltd., United Kingdom) in conjunction with the Breathelink feedback system.

**FIGURE 2:**
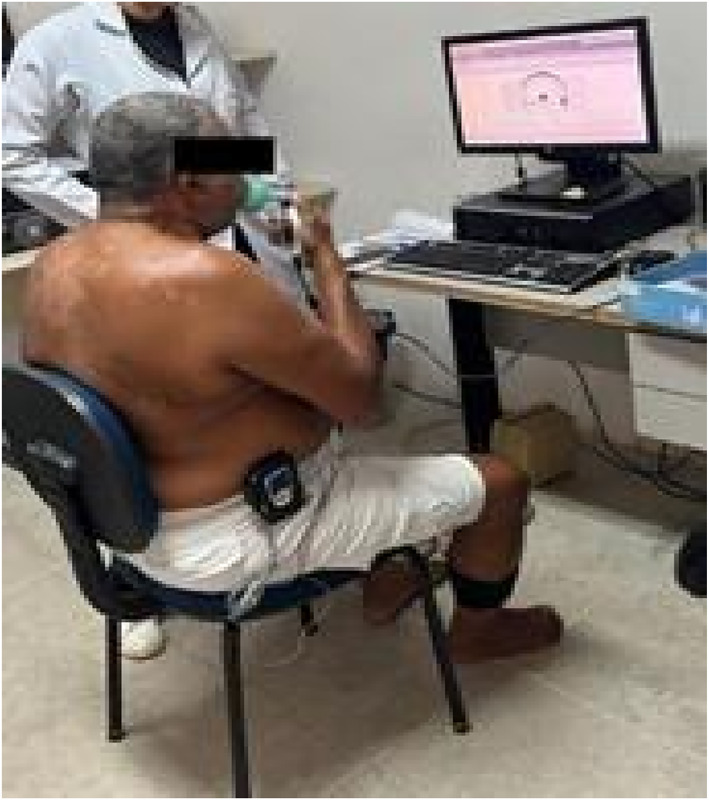
Participant performing IMT with NIRS monitoring coupled to the medial gastrocnemius muscle. *Source:* Personal Archive. IMT: Inspiratory muscle training; NIRS: Near‐Infrared Spectroscopy.

Participants in the strength‐based IMT group performed inspiratory muscle training consisting of three sets of 1 min of maximal and deep inspirations with 2 min of rest between sets using a load equivalent to 60% of the MIP determined during their initial assessment. Participants in the endurance‐based IMT group performed three sets of 2 min of maximal and deep inspirations with 2 minutes of rest between sets, using a load corresponding to 30% of their baseline MIP (Weiner and McConnell 2005). Participants in the sham group performed three sets of 2 minutes of maximal and deep inspirations with the device set at the minimal load (3 cmH_2_O) followed by 2 min of rest between sets (Figure 2).

The calibration of the KH2 device was performed before each session, including verification of the mouthpiece seal, adjustment of resistance according to the individual MIP, and equipment testing to ensure measurement accuracy. Adjustments and calibrations were conducted to guarantee the reproducibility of the protocol. The frequency and duration of the sessions followed the experimental study protocol, ensuring fidelity in the application of the interventions. This level of detail follows the TIDieR recommendations, allowing the protocol to be replicated in other respiratory rehabilitation studies.

All sessions were conducted individually, under the supervision of physiotherapists trained in the protocol, and visual feedback provided by the Breathelink system was used to ensure proper execution. During the study, mild adverse effects, including dyspnea and dizziness, were observed in 10% of participants. No serious adverse events were reported.

### 2.6 Outcomes

The rate of peripheral muscle oxygen desaturation was defined as the primary outcome of the study. This variable reflects the rate at which oxygen saturation in muscle tissue decreases per unit of time during the performance of IMT exercises.

The assessment was conducted using NIRS (Artinis, Portamon system, The Netherlands), a noninvasive method that allows continuous and real‐time monitoring of tissue oxygenation. The desaturation rate is a sensitive indicator of the oxidative capacity of peripheral muscles and may provide relevant information regarding the acute effects of IMT on muscle physiology in individuals with COPD. Was calculated as the difference between baseline tissue oxygen saturation and the lowest StO_2_ value reached during the protocol, divided by the time required to reach this minimum value.

The NIRS device was connected via Bluetooth to a computer, enabling continuous recording of variables and real‐time feedback through the Oxysoft software (Artinis Medical Systems BV 2012). During the evaluations, participants remained seated with knees flexed at 90°, and the assessed limb was immobilized to avoid signal interference.

During IMT in the three experimental interventions, peripheral muscle metabolism was simultaneously evaluated using NIRS. The initial saturation, final saturation, delta saturation of the peripheral musculature and oxygen desaturation rate according to GOLD severity groups were considered secondary outcomes.

### 2.7 Statistical Analysis

Data were initially organized in Excel and subsequently exported to SPSS software, version 25, for statistical analysis. The normality of the data was verified using the Shapiro–Wilk test. Descriptive statistics were used to characterize the sample with values expressed as mean and standard deviation (SD).

The main inferential analysis aimed to compare the effects of the three experimental conditions (IMT‐Strength, IMT‐Endurance, and Sham) on the dependent variables (oxygen saturation, oxygen desaturation time, and oxygen desaturation rate). Linear mixed models with interaction terms (group and time) were used to analyze intra‐ and intergroup differences in peripheral tissue oxygen saturation variables. Group and time were treated as fixed factors, while participants were considered random factors. In addition, the Q–Q plots confirmed the residual normality for all analyzed outcomes.

Oxygen desaturation time and desaturation rate were analyzed using repeated‐measures Analysis of Variance (ANOVA). The application of this test followed verification of the sphericity assumption using Mauchly's test. When ANOVA indicated significant differences, the Least Significant Difference (LSD) post hoc test was applied for pairwise comparisons between interventions.

In the second stage, a secondary analysis was conducted to explore the impact of disease severity on the observed outcomes. Participants were stratified into two distinct groups based on the GOLD classification: a lower‐severity group (GOLD 1 and 2) and a higher‐severity group (GOLD 3 and 4). To compare the mean values of the variables of interest between these two independent groups, the independent samples *t*‐test was applied. The assumption of homogeneity of variances, essential for the validity of the *t*‐test, was previously assessed using Levene's test. All data were analyzed with a 5% level of significance (*p* < 0.05) and a 95% confidence interval (95% CI).

## 3 Results/Findings

**FIGURE 3:**
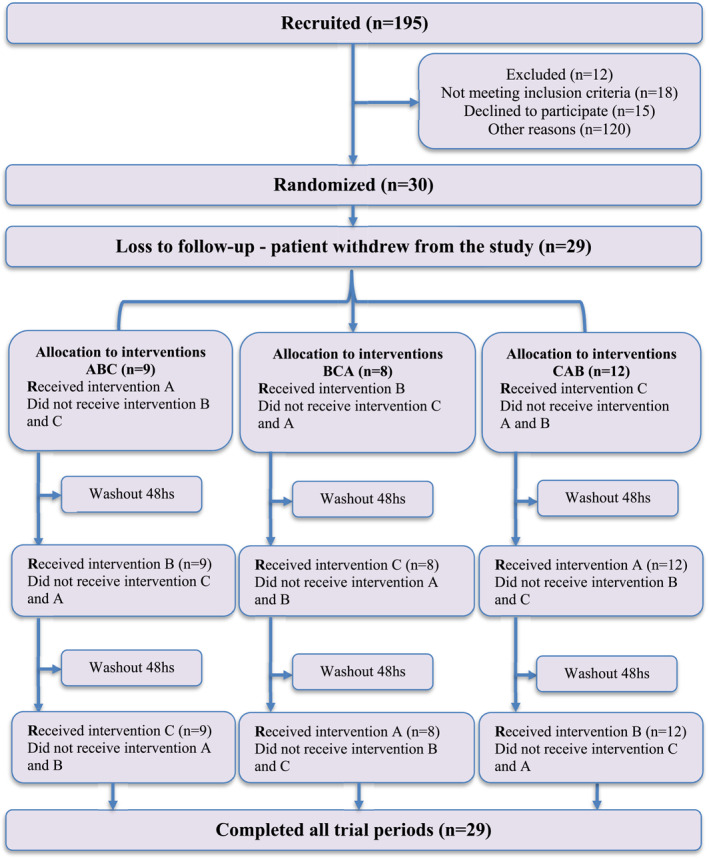
CONSORT flow diagram for crossover trials. A: IMT‐Strength; B: IMT‐Endurance; C: Sham; CONSORT: Consolidated Standards of Reporting Trials.

One hundred ninety‐five participants were recruited for the research, but 165 were not included because: they did not respond to contact, refused to participate, did not meet the eligibility criteria, or for other reasons. Thus, 30 participants remained who were randomized into different intervention orders with 1 participant dropping out before completing the sessions. Therefore, the final sample consisted of 29 participants (Figure 3).

The study sample was composed of 29 individuals with COPD, predominantly of mixed ethnicity, with a slight predominance of males and an average age compatible with an elderly population, with a significant history of smoking. A predominance of stages 1 and 2 of the GOLD classification was observed. The participants presented a distance covered in the 6MWT lower than the predicted value at 369.21 m, MIP below the predicted value at 56.19 cmH2O, demonstrating inspiratory muscle weakness (ATS/ERS Statement on respiratory muscle testing, 2002), and pulmonary function with an obstructive ventilatory disorder, evidenced by reductions in FEV1 with a mean value of 1.41 L and FEV1/FVC of 57% (Table 1).

**TABLE 1:** Demographic, clinical, and lung function characteristics of the study subjects (*n* = 29).

Variable	All subjects (*n* = 29)	Predicted values
Male/Female, *n*	16/13	—
White/Mixed, *n*	9/20	—
Age, years	66.17 ± 9.97	—
GOLD 1/2/3/4	7/13/6/3	—
6MWT distance, meters	369.21 ± 154.34	595.24 ± 59.51
MIP, cmH_2_O	56.19 ± 22.66	92,41 ± 14.84
Post‐bronchodilator pulmonary function		
FEV1, liters	1.41 ± 0.71	5.15 ± 2.25
FVC, liters	2.40 ± 0.93	6.89 ± 2.15
FEV1/FVC, %	57 ± 11.2	71.76 ± 13.85

Abbreviations: 6MWT: 6‐min walk test; FEV1/FVC, Ratio of FEV1 to FVC; FEV_1_: forced expiratory volume in one second; FVC: forced vital capacity; GOLD: Global Initiative for Chronic Obstructive Lung Disease; L/s: liters per second; MIP: Maximum inspiratory pressure; *n*: number of individuals.

Table 2 presents the estimated mean, standard error, and confidence interval of initial and final oxygen saturation (O_2_). It also shows the median and confidence interval of the oxygen (O_2_) desaturation time, as well as the mean and standard deviation of the O_2_ desaturation rate, measured by NIRS during the strength, endurance, and Sham IMT protocols. An increase in final O_2_ saturation compared with initial values was observed only in the endurance IMT group, while a significant reduction in O_2_ desaturation time was found in the group subjected to the strength IMT protocol compared with the endurance and Sham groups.

**TABLE 2:** Values of peripheral tissue oxygen saturation, oxygen desaturation time, and desaturation rate in the different IMT protocols in individuals with COPD.

Protocol	Time (moment)	Estimated mean (%)	Standard deviation	95% CI lower	95% CI upper	p‐MIXED
IMT‐strength	Initial StO_2_	55.76	6.35	53.33	58.20	0.133
	Final StO_2_	56.33	6.55	53.89	58.77	
IMT‐endurance	Initial StO_2_	55.62	6.17	53.18	58.06	0.003*
	Final StO_2_	56.76	6.18	54.32	59.20	
Sham	Initial StO_2_	55.88	6.59	53.44	58.32	0.115
	Final StO_2_	56.48	6.36	54.04	58.92	

Variable	Sham M(IQ)	IMT‐strength M(IQ)	IMT‐endurance M(IQ)	gl	*η* ^2^	*p*‐ANOVA
Desaturation time (s)	255.1 (59.1–511.1)	59.4 (29–392.9)	88.7 (40.7–405.5)	2	0.054	**0.021**
O_2_ desaturation rate (%/s)	0.005 (0.001–0.014)	0.006 (0.003–0.014)	0.008 (0.002–0.016)	2	0.010	0.397

*Note:* The values represent the estimated means and their respective standard errors obtained using a linear mixed model with compound symmetry (CS) covariance structure and the restricted maximum likelihood (REML) method. Multiple comparisons were adjusted using the Bonferroni method. Oxygen (O_2_) desaturation rate and time were analyzed using ANOVA, considering the Greenhouse–Geisser correction for sphericity and *η* ^2^ for effect size, with variables reported as median and interquartile range (25th–75th percentile).

Abbreviations: IMT: inspiratory muscle training; StO_2_, Tissue oxygen saturation; O2.

The mixed linear model analysis demonstrated a significant main effect of the Time factor on gastrocnemius muscle tissue oxygen saturation (StO_2_) (F (1.84) = 12.619; *p* = 0.001). This finding indicates that, regardless of the protocol applied, there was a significant change in StO_2_ across assessments, reflecting an overall increase in muscle oxygenation from pre‐to post‐intervention.

In contrast, no main effect of the Group factor was identified (F (2.84) = 0.005; *p* = 0.995), suggesting that the different inspiratory muscle training (IMT) protocols did not differ in terms of mean StO_2_ levels when considered independently. Furthermore, the absence of a significant Time × Group interaction (F (2.84) = 0.741; *p* = 0.480) indicates that the magnitude and direction of changes in muscle oxygenation over time were similar among groups, with no evidence that a specific protocol promoted superior adaptations compared with the others.

In the within‐group comparisons, only the IMT‐Endurance group showed a significant increase in StO_2_ from baseline (55.62) to post‐intervention (56.76; *p* = 0.003), whereas the changes observed in the IMT‐Strength group (55.76–56.33; *p* = 0.133) and the Sham group (55.88–56.48; *p* = 0.115) did not reach statistical significance.

In the between‐group analysis, a difference was observed in gastrocnemius muscle oxygen desaturation time (*p* = 0.021; *η* ^2^ = 0.054), specifically during the IMT‐Strength protocol. The Sham group exhibited the longest median desaturation time (median = 255.10 s; IQR = 59.1–511.10 s), followed by the IMT‐Endurance group (median = 88.70 s; IQR = 40.7–405.50 s) and the IMT‐Strength group (median = 59.40 s; IQR = 29–392.90 s).

Conversely, the oxygen (O_2_) desaturation rate showed no significant differences among the protocols (*p* = 0.397; *η* ^2^ = 0.010), with mean values of 0.005 (0.001–0.014) in the Sham group, 0.006 (0.003–0.014) in the IMT‐Strength group, and 0.008 (0.002–0.016) in the IMT‐Endurance group.

To investigate whether the severity of COPD could alter the tissue oxygen desaturation rate in the three proposed interventions, the participants were divided into two subgroups: mild to moderate severity (GOLD 1 and 2), and severe to very severe (GOLD 3 and 4). We observed that there was no difference in the tissue oxygen desaturation rate in all proposed interventions, regardless of the severity of COPD. However, during the strength IMT intervention, individuals classified as GOLD 3–4 exhibited a numerically higher tissue oxygen desaturation rate compared with those classified as GOLD 1–2 (Table 3); however, this difference was descriptive in nature and did not reach statistical significance.

**TABLE 3:** Comparison of the desaturation rate (%/s), in relation to COPD severity in the three studied groups.

Severity of COPD	Sham (mean ± SD)	IMT‐strength (mean ± SD)	IMT‐endurance (mean ± SD)
Desaturation rate (%/s) GOLD 1 and 2	0.007 ± 0.012	0.012 ± 0.012	0.021 ± 0.035
Desaturation rate (%/s) GOLD 3 and 4	0.014 ± 0.016	0.082 ± 0.254	0.009 ± 0.009
*p*‐value	0.179	0.267	0.225

Abbreviations: COPD: Chronic Obstructive Pulmonary Disease; GOLD: Global Initiative for Chronic Obstructive Lung Disease; IMT‐Endurance: Endurance Inspiratory Muscle Training; IMT‐Strength: Strength Inspiratory Muscle Training; SD: Standard Deviation; Sham: Control intervention protocol.

## 4 Discussion

Our study demonstrated that peripheral tissue oxygenation increased in individuals with COPD during endurance IMT. When analyzing tissue O_2_ desaturation time and rate, we observed that during strength IMT, the time required to reach the lowest tissue O_2_ saturation was shorter than in the other protocols. As a result, the calculated desaturation rate was higher. Additionally, individuals classified as GOLD 3–4 exhibited a tissue O_2_ desaturation rate nearly eight times higher than those classified as GOLD 1–2 during strength IMT. Importantly, these findings should be interpreted as reflecting acute physiological responses to different inspiratory loads, rather than indicating clinically relevant tissue hypoxia or risk to peripheral muscles.

Regarding sample characterization and the predominance of male participants, our findings are consistent with those reported by Wang et al. (2023), who described exercise‐induced oxygen desaturation in individuals with COPD during the six‐minute walk test (6MWT). In our study, participants walked an average of 369 m in the 6MWT, a distance close to the lower threshold of 350 m. This threshold has been associated with an increased risk of exacerbations, hospitalizations, and mortality in COPD. Inspiratory muscle weakness, also identified in our cohort, is a known contributor to dyspnea, fatigue, and reduced quality of life in this population (Mota et al. 2023).

The observed changes in tissue O_2_ saturation, as well as in desaturation time and rate during strength and endurance IMT, likely represent the manifestation of a complex and transient physiological response to the imposed inspiratory load (Fernández‐Rubio et al. 2021). Differences in load magnitude, intervention duration, and ventilator demand across IMT protocols may influence systemic hemodynamic adjustments and autonomic regulation, thereby modulating peripheral blood flow and oxygen delivery. In the absence of direct activation of the locomotor muscles, these responses are more likely driven by systemic and neural mechanisms rather than by local metabolic demand.

Previous studies investigating peripheral oxygenation during respiratory muscle loading have primarily focused on acute responses. Borghi‐Silva et al. (2008) demonstrated improved leg muscle oxygenation when respiratory muscle workload was reduced during exercise. More recent experimental studies have reported variable acute tissue O_2_ responses following IMT sessions, depending on the type and intensity of the inspiratory stimulus. Collectively, these findings suggest that IMT can influence peripheral oxygenation acutely, although such effects are multifactorial and dependent on training load, duration, disease severity, and baseline oxygenation status.

The inspiratory muscle metaboreflex has been proposed as one of the mechanisms linking respiratory muscle work to peripheral oxygenation. As described by Lima et al. (2025), activation of this reflex may occur when inspiratory muscles are exposed to high metabolic stress, leading to sympathetic‐mediated vasoconstriction in non‐respiratory muscles (Witt et al. 2007). However, it is important to emphasize that activation of the inspiratory metaboreflex is not an all‐or‐none phenomenon and may occur in a subtle, load‐dependent manner, particularly during brief or isolated inspiratory efforts. In this context, the modest changes observed in peripheral tissue oxygenation in our study do not necessarily indicate harmful or clinically significant desaturation.

In our study, endurance IMT was the only protocol associated with an increase in peripheral tissue oxygenation, which may be explained by its lower load and reduced likelihood of inducing inspiratory muscle fatigue and subsequent metaboreflex activation. Conversely, the shorter tissue O_2_ desaturation time observed during strength IMT may reflect a more intense acute inspiratory workload, capable of eliciting stronger autonomic adjustments, rather than a pathological reduction in muscle oxygen supply (Fernández‐Rubio et al. 2021).

When stratified by disease severity, individuals classified as GOLD 3–4 exhibited a markedly higher desaturation rate during strength IMT, although this difference did not reach statistical significance. This finding may be related to the greater mechanical disadvantage, dynamic hyperinflation, and reduced inspiratory reserve commonly observed in more advanced COPD. These factors may predispose individuals to earlier physiological stress during high‐load inspiratory efforts. Nevertheless, these responses should be interpreted within the framework of acute physiological modulation, rather than as indicators of tissue damage or clinical risk.

Moreover, in advanced COPD, peripheral muscle oxygen utilization may be limited by intrinsic muscular alterations rather than by oxygen delivery alone. Structural and functional abnormalities—such as fiber‐type shifts, capillary rarefaction, and mitochondrial dysfunction—reduce oxidative capacity and may create a physiological ceiling effect for oxygen extraction (Layec et al. 2017; Jaitovich and Barreiro 2018). Under these conditions, modest acute fluctuations in tissue oxygenation are unlikely to translate into clinically meaningful consequences.

Thus, the in vivo functional findings of the present study align with previous evidence demonstrating that peripheral myopathy is a key determinant of exercise limitation in COPD (Gökmen and Demir 2023). While long‐term IMT has been shown to attenuate inspiratory metaboreflex activation and improve exercise tolerance (Lima et al. 2025), the present investigation focused exclusively on acute responses, which should not be extrapolated to chronic adaptations.

Several methodological considerations must be acknowledged. The cross‐sectional design allowed assessment of only a single session of each IMT modality, and acute physiological responses should be clearly distinguished from long‐term training adaptations. Additionally, individual variability in physical conditioning and the absence of adjustment for potential confounders may have influenced the observed responses.

### 4.1 Implications for Physiotherapy Practice

In conclusion, a single IMT session was sufficient to induce acute, load‐dependent changes in peripheral tissue oxygenation in individuals with COPD. Strength IMT was associated with a shorter time to tissue O_2_ desaturation, particularly in individuals with more advanced disease. However, these responses were modest and should not be interpreted as clinically dangerous or indicative of peripheral muscle hypoxia. Rather, they reflect transient physiological adjustments to inspiratory loading.

Therefore, our findings underscore the importance of individualized IMT prescription in COPD, taking disease severity into account, while also reinforcing that the observed acute changes do not imply clinical risk. These results provide a physiological basis for optimizing IMT strategies within pulmonary rehabilitation. This approach aims to maximize efficacy while maintaining safety.

## Author Contributions

All authors contributed equally to the manuscript and read and approved the final version of the manuscript.

## Funding

This study received financial support from the National Council for Scientific and Technological Development (CNPq)/MCTI, Call No. 10/2023 — Track A—Emerging Group. Grant No: 404976/2023‐9.

## Ethics Statement

The study was approved by the Ethics and Research Committee of the Health Sciences Center of the Federal University of Paraíba (CAAE: 75362823.4.0000.5188).

## Consent

All participants signed the informed consent form following resolution 466/12 of the Brazilian National Health Council and Declaration of Helsinki. Consents were obtained by the patient of the cases.

## Conflicts of Interest

The authors declare no conflicts of interest.

## Data Availability

The data that support the findings of this study are available from the corresponding author upon reasonable request.
